# Prognostic value of OCT biomarkers and systemic factors for early and long term anatomical treatment response in diabetic macular edema

**DOI:** 10.1186/s12886-026-05350-2

**Published:** 2026-09-17

**Authors:** Tuğçe Özdemir, Gürkan Erdoğan, Zafer Cebeci

**Affiliations:** 1 Department of Ophthalmology, Korgan State Hospital, 11A Bağ Street, Dolunay Neighborhood, Fatsa, Ordu Turkey; 2 Department of Ophthalmology, Istanbul Faculty of Medicine, Istanbul University, Istanbul, Turkey

**Keywords:** Diabetic macular edema, Anti-VEGF therapy, OCT biomarkers, Anatomical response, Hyperreflective intraretinal material, Hyperreflective foci

## Abstract

**Background:**

To evaluate the prognostic value of baseline optical coherence tomography (OCT) biomarkers and systemic factors for early and long-term anatomical response to anti-VEGF therapy in diabetic macular edema (DME).

**Methods:**

This retrospective study included 164 eyes from 105 patients with type 2 diabetes and DME who received three monthly loading doses of intravitreal bevacizumab (January 2013 to April 2024) and were subsequently managed with a pro re nata (PRN) regimen using standard retreatment criteria. Completion of the loading phase (minimum follow-up, 3 months) was required for inclusion. Anatomical response was assessed after the loading phase (early response) and at final follow-up (long-term response); eyes were classified as full, partial, or non-responders based on CMT and OCT morphology. Baseline demographic, systemic, and OCT characteristics were analyzed using generalized estimating equation (GEE) logistic regression to account for inter-eye correlation, followed by one-eye-per-patient sensitivity analyses.

**Results:**

After loading, 10 eyes (6.1%) were full responders, 72 (43.9%) partial responders, and 82 (50.0%) non-responders; 90% of early full responders maintained full response at final follow-up. In multivariable GEE analyses, higher baseline CMT, higher hyperreflective foci (HRF) count, and hyperreflective intraretinal material were independently associated with early anatomical response under the original percentage-reduction outcome definition; for long-term response, baseline CMT was only nominally associated and did not survive correction for multiple testing, and DRIL, EZ/ELM defect, and intraretinal cyst size were not significant. Both models showed moderate discrimination after bootstrap optimism correction (AUC 0.751 and 0.757, respectively). Sensitivity analyses across eye-selection strategies were consistent for baseline CMT, DRIL, EZ/ELM defect, and hyperreflective material, but intraretinal cyst size was unstable; all associations were sensitive to how anatomical response was defined.

**Conclusions:**

Baseline OCT biomarkers showed candidate associations with anatomical response, but several, including the hyperreflective-material association, were not reproduced when the outcome was redefined using continuous final CMT or an absolute CMT threshold, despite robustness within the primary model. Discrimination was moderate (optimism-corrected AUC 0.75–0.76). These findings identify candidate prognostic OCT biomarkers requiring external validation before clinical application.

**Supplementary Information:**

The online version contains supplementary material available at https://doi.org/10.1186/s12886-026-05350-2.

## Introduction

Diabetic macular edema (DME) is the leading cause of vision loss in patients with diabetic retinopathy (DR) and is increasing in parallel with the global epidemic of type 2 diabetes [1–3]. The prevalence of DME in patients with DR has been reported to range between 2.7% and 11% [4–6]. The pathophysiological process begins with hyperpermeability caused by pericyte and endothelial cell loss in retinal capillaries. This vascular damage results in retinal ischemia, triggering the release of various mediators—among which vascular endothelial growth factor (VEGF) is considered the most critical. Elevated VEGF levels, particularly in cases of worsening ischemia, are one of the primary factors contributing to the development of macular edema [7]. Although VEGF plays a key role, the pathophysiology of DME is multifactorial, involving inflammatory pathways, ischemia, and neurodegenerative processes.

While the foundation of DME treatment lies in achieving good glycemic control, intravitreal injection of anti-VEGF agents is considered the first-line treatment due to their superior anatomical and functional outcomes [8]. However, approximately 25% of patients exhibit suboptimal response to anti-VEGF therapy [9–11]. Early responses to anti-VEGF therapy in DME may provide valuable insights into long-term treatment outcomes. This facilitates timely adjustments in treatment strategies, including switching between therapeutic agents or combining regimens when necessary [12, 13].

Optical coherence tomography (OCT) is currently the most important tool for evaluating and monitoring treatment response in DME. OCT allows identification of structural biomarkers such as intraretinal fluid, intraretinal cysts (IRCs), retinal layer disruptions, hyperreflective foci (HRF), and vitreoretinal interface abnormalities, all of which offer valuable insights into the disease course and response to therapy in diabetic retinopathy [14].

There is limited literature simultaneously evaluating the relationship between retinal OCT biomarkers and systemic factors with the treatment response in DME. In this study, we aimed to investigate baseline OCT biomarkers and systemic parameters affecting anatomical response to three consecutive intravitreal bevacizumab injections (loading dose) and to long-term anatomical prognosis, and to descriptively compare the pattern of early and long-term anatomical response.

## Materials and methods

This retrospective study was approved by the Clinical Research Ethics Committee of Istanbul Faculty of Medicine, Istanbul University (Approval No. 2024/514; March 8, 2024), and was conducted in accordance with the tenets of the Declaration of Helsinki. This study is reported in accordance with the STROBE (Strengthening the Reporting of Observational Studies in Epidemiology) statement for observational cohort studies and, given its prognostic framing, the TRIPOD (Transparent Reporting of a multivariable prediction model for Individual Prognosis Or Diagnosis) statement for studies developing a multivariable prediction model without external validation.

A total of 164 eyes from 105 patients with type 2 diabetes mellitus diagnosed with DME who received three consecutive monthly loading doses of intravitreal bevacizumab between January 2013 and April 2024 at the Retina Department of the Ophthalmology Clinic, Istanbul Faculty of Medicine, were included in this study. Following completion of the loading phase, patients were treated according to a pro re nata (PRN) regimen. Additional intravitreal anti-VEGF therapy (bevacizumab, ranibizumab, or aflibercept) was administered in the presence of persistent or recurrent intraretinal and/or subretinal fluid on OCT, an increase in central macular thickness (CMT) compared with the previous visit, or deterioration in best-corrected visual acuity (BCVA) that could not be explained by other ocular causes.

Eyes with previous intravitreal corticosteroid treatment, incomplete loading therapy, loading injections not administered at approximately one-month intervals, inadequate follow-up, poor-quality OCT images precluding reliable evaluation, previous vitreoretinal surgery, epiretinal membrane, vitreomacular traction or vitreomacular adhesion, and concomitant retinal diseases affecting macular morphology or visual acuity were excluded. All exclusion criteria, including vitreoretinal interface abnormalities, were applied during the original data extraction, and no eyes were subsequently removed during manuscript revision; the analyzed sample remained 164 eyes from 105 patients throughout all revisions. A numerical accounting of the number of eyes excluded for each specific criterion at the point of data extraction is not available and is stated as a limitation. Completion of the three-injection loading phase, corresponding to a minimum follow-up of 3 months, was required for inclusion. The observed minimum follow-up in the analyzed cohort was 6 months rather than 3 months; because inadequate follow-up was a separate exclusion criterion applied at the time of data extraction, no eye with follow-up in the 3–6 month range concurrently satisfied both the loading-phase-completion requirement and the adequate-follow-up requirement in this cohort, even though 3 months remained the minimum formally required for loading-phase completion.

### Patient grouping and definitions

Based on the anatomical response after completion of the three loading injections (early response) and at the final follow-up visit (long-term response), eyes were classified into three predefined response categories. Full responders were defined as eyes demonstrating complete resolution of intraretinal and/or subretinal fluid with restoration of a normal foveal contour on OCT. Partial responders were defined as eyes demonstrating a ≥ 20% reduction in CMT compared with baseline but with persistent macular edema and incomplete restoration of the foveal contour. Non-responders were defined as eyes demonstrating < 20% reduction in CMT compared with baseline. The ≥ 20% reduction threshold was selected based on previous studies evaluating early anatomical response to anti-VEGF therapy in DME [15, 16].

Because the primary objective of the multivariable analysis was to identify independent predictors of anatomical response, an additional binary outcome (anatomical responder vs. non-responder, defined as ≥ 20% reduction in CMT from baseline) was created separately for the post-loading and final follow-up visits for the generalized estimating equation (GEE) analyses. The percentage-reduction responder definition was retained as the primary outcome to preserve comparability with previous DME responder studies and because it provides a clinically interpretable binary classification. Continuous final CMT and an absolute CMT threshold were examined as sensitivity outcomes because they are not mathematically coupled to baseline CMT.

This classification was based on anatomical OCT findings, as structural improvement represents the primary outcome measure in the evaluation of DME treatment response. Functional outcomes, including BCVA, were analyzed separately to assess the relationship between anatomical and functional responses.

Baseline demographic characteristics (age, sex, duration of follow-up, duration of diabetes mellitus, hypertension), systemic variables (HbA1c, lipid profile, and diabetes treatment modality), ophthalmic findings (BCVA and fundus examination), and OCT biomarkers were retrospectively reviewed and compared among the response groups. BCVA was measured using Snellen visual acuity charts and converted to the logarithm of the minimum angle of resolution (logMAR) for statistical analysis. Snellen-derived logMAR values are less granular than ETDRS letter-based measurements, particularly at the extremes of visual acuity, which is noted as a limitation. Missing data varied by variable among the 106 patients for whom baseline systemic data were extracted (one of whom was subsequently excluded from the final analytic sample of 105 patients per the exclusion criteria above), with duration of diabetes missing for 13/106 patients (12.3%), hypertension status for 7/106 (6.6%), baseline and final HbA1c for 34/106 (32.1%) and 35/106 (33.0%) respectively, and total cholesterol and LDL cholesterol for 68/106 each (64.2%); all other demographic, ocular, and OCT variables were missing for fewer than 1% of patients or eyes. Univariable and multivariable GEE analyses used all eyes with complete data for the variables included in that specific model (complete-case analysis), so that the effective sample size differs across models and is reported alongside each result; no imputation was performed.

### OCT biomarkers and imaging parameters

All OCT examinations were performed using spectral-domain OCT (Heidelberg Engineering, Heidelberg, Germany). OCT images were independently evaluated by two experienced ophthalmologists who were masked to the clinical outcomes. Any disagreement was resolved by consensus.

CMT was defined as the mean retinal thickness within the central 1-mm ETDRS subfield. Subfoveal choroidal thickness (SFCT) was measured manually from the outer border of the retinal pigment epithelium to the inner scleral surface using enhanced-depth imaging mode.

Hard exudates were defined as hyperreflective lesions measuring > 30 μm with posterior shadowing and reflectivity similar to the retinal pigment epithelium–Bruch’s membrane complex.

**Fig. 1:**
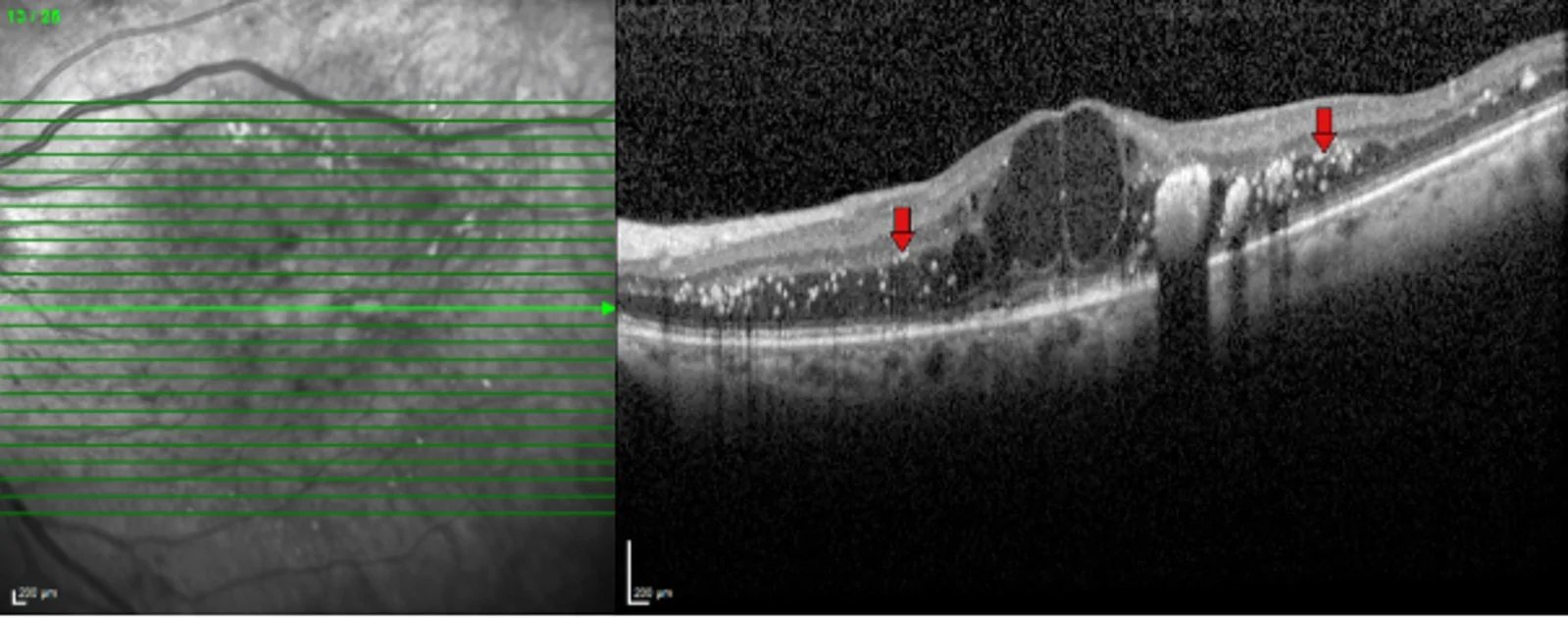
Representative spectral-domain OCT image demonstrating multiple intraretinal hyperreflective foci in an eye with diabetic macular edema (arrows)

HRF were defined as discrete, round hyperreflective lesions measuring < 30 μm without posterior shadowing and located within any retinal layer and were manually counted within the central 3-mm area (Fig. 1).

**Fig. 2:**
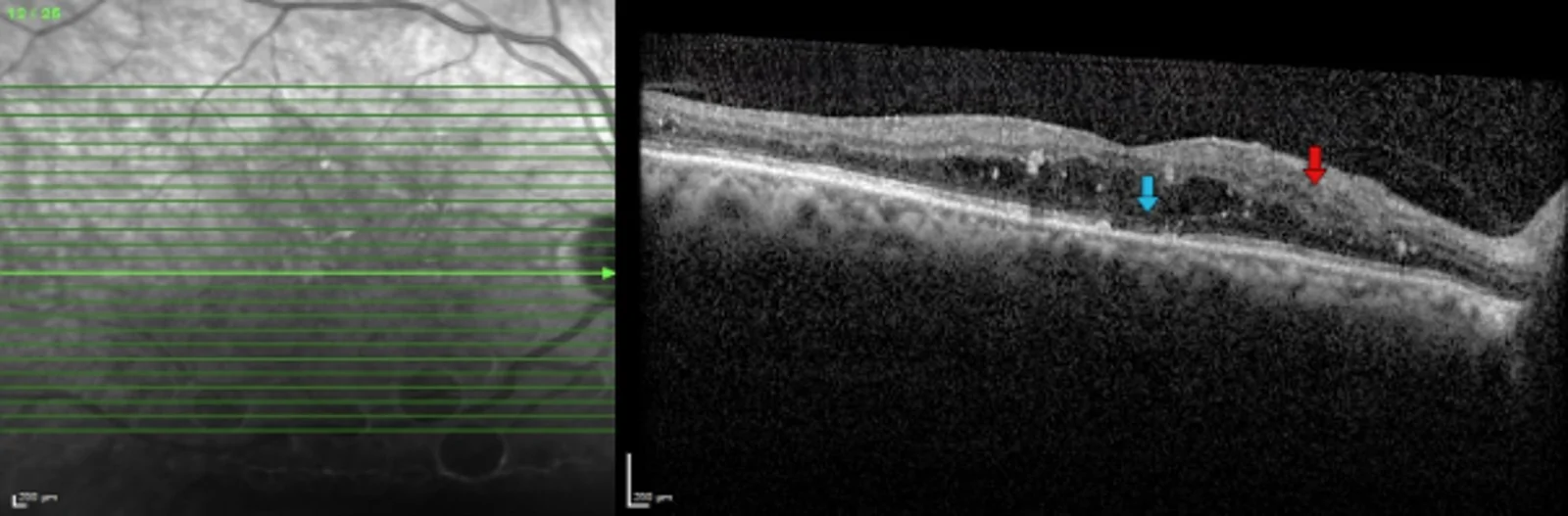
Representative spectral-domain OCT image demonstrating DRIL (red arrow) and EZ/ELM disruption (blue arrow) in an eye with diabetic macular edema

Disorganization of the retinal inner layers (DRIL) was defined as indistinguishable boundaries between the ganglion cell layer, inner plexiform layer, inner nuclear layer, and outer plexiform layer within the central 1-mm zone. Ellipsoid zone/external limiting membrane (EZ/ELM) defects were defined as discontinuity of either the ellipsoid zone or the external limiting membrane within the central 1-mm area (Fig. 2).

**Fig. 3:**
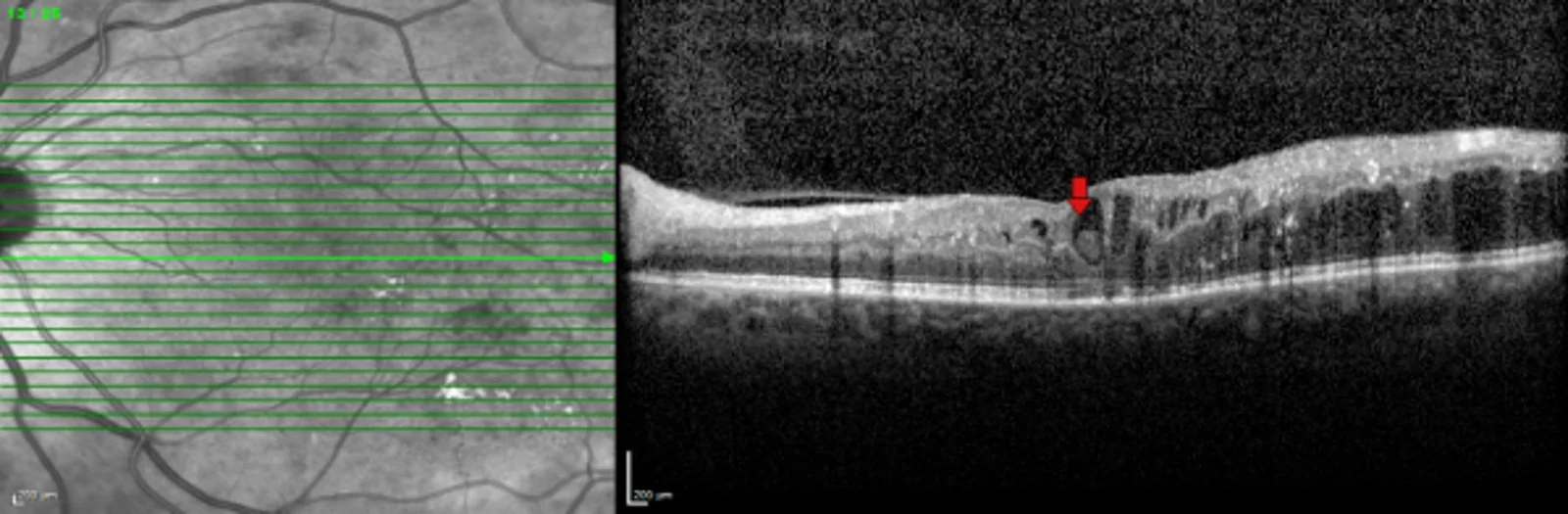
Representative spectral-domain OCT image demonstrating hyperreflective intraretinal material within intraretinal cystoid spaces in diabetic macular edema (arrow)

Hyperreflective intraretinal material was defined as hyperreflective material within the cyst lumen exhibiting reflectivity similar to the surrounding retinal tissue (Fig. 3).

**Fig. 4:**
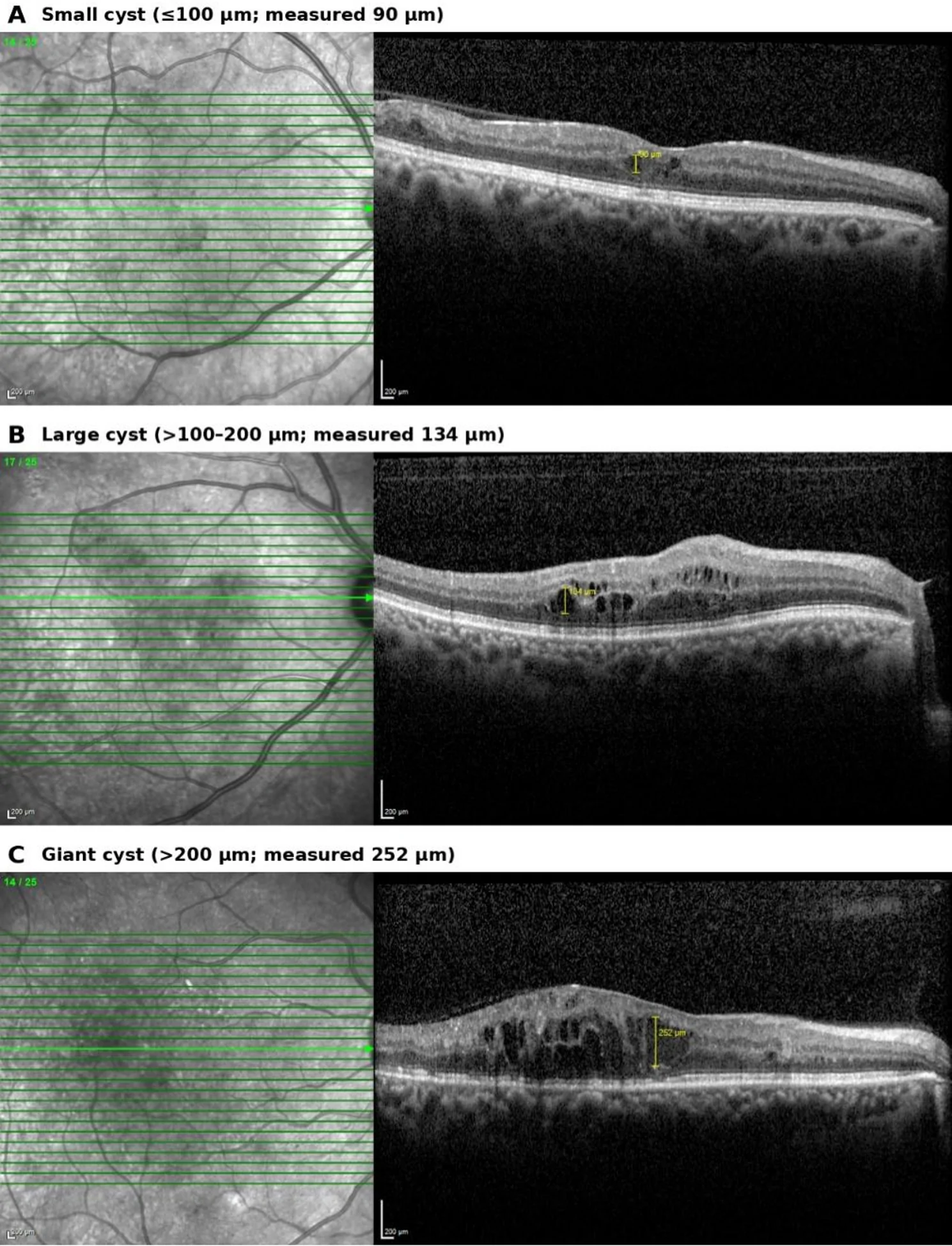
Representative spectral-domain OCT images illustrating the three intraretinal cyst-size categories used in this study, with the infrared fundus image and scan location shown alongside each B-scan and the vertical caliper measurement displayed on the image. (**A**) Small cyst (≤ 100 μm; measured 90 μm). (**B**) Large cyst (> 100–200 μm; measured 134 μm). (**C**) Giant cyst (> 200 μm; measured 252 μm). Scale bars, 200 μm

Intraretinal cysts were defined as hyporeflective round or oval spaces separated by hyperreflective septa. The largest vertical cyst diameter within the central 1-mm area was measured manually and classified as small (≤ 100 μm), large (> 100–200 μm), or giant (> 200 μm) according to previously published OCT-based classifications of diabetic macular edema [17, 18] (Fig. 4); these specific numerical cut-offs are stated verbatim in reference 17 (Suciu et al.), which classifies cysts as small (< 100 μm), large (101–200 μm), and giant (> 200 μm).

Because SFCT and cyst size measurements were performed manually, a certain degree of measurement variability is inevitable and should be considered when interpreting the findings. To assess inter-observer reproducibility, the two original masked observers independently re-graded baseline images for a randomly selected subset of 30 eyes (fixed random seed = 2026), presented in a shuffled, de-identified order with no access to follow-up images or knowledge of outcome status; agreement for DRIL, EZ/ELM defect, hyperreflective intraretinal material, and cyst-size category was quantified using Cohen’s kappa, and agreement for HRF count and SFCT was quantified using the intraclass correlation coefficient and Bland-Altman limits of agreement (Supplementary Table S10).

### Statistical analysis

Statistical analyses were performed using SPSS version 27.0 (IBM Corp., Armonk, NY, USA). Continuous variables are presented as mean ± standard deviation (SD), whereas categorical variables are presented as frequencies and percentages. Data distribution was assessed using the Shapiro–Wilk test. Depending on the distribution of the data, paired t-test, one-way analysis of variance (ANOVA), Wilcoxon signed-rank test, Kruskal–Wallis test, or Friedman test was applied to the descriptive group comparisons in Tables 1 and 2. For the within-eye longitudinal comparisons across baseline, post-loading, and final follow-up (Table 3), a naive paired test would not account for the correlation between the two eyes of the same patient; these comparisons were therefore performed using a repeated-measures GEE model (Gaussian family, identity link, exchangeable working correlation, clustered by patient, with time modeled as a three-level categorical factor), and pairwise time-point contrasts are reported from this model rather than from separate paired t-tests. Categorical variables were compared using the chi-square test or Fisher’s exact test, as appropriate. A p-value < 0.05 was considered statistically significant for descriptive analyses.

**Table 1:** Baseline clinical and OCT characteristics according to early anatomical response

Parameter	Full (*n* = 10)	Partial (*n* = 72)	Non (*n* = 82)	*p*
CMT (µm)	345.6 ± 97.1	506.9 ± 143.5	428.9 ± 126.6	0.001
BCVA (logMAR)	0.37 ± 0.44	0.48 ± 0.44	0.40 ± 0.36	0.495
SFCT (µm)	305.5 ± 45.8	326.9 ± 95.3	307.3 ± 84.6	0.366
HRF	5.2 ± 7.0	8.8 ± 6.7	7.3 ± 5.1	0.110
DRIL n (%)	5 (50.0%)	58 (80.6%)	47 (57.3%)	0.004
EZ/ELM defect n (%)	2 (20.0%)	46 (63.9%)	38 (46.3%)	0.011
Cyst size				0.002
Small cyst (≤ 100 μm)	7 (70.0%)	8 (11.1%)	16 (19.5%)	
Large cyst (> 100–200 μm)	1 (10.0%)	12 (16.7%)	22 (26.8%)	
Giant cyst (> 200 μm)	2 (20.0%)	52 (72.2%)	44 (53.7%)	
Hyperreflective intraretinal material	3 (30.0%)	16 (22.2%)	12 (14.6%)	0.310

Continuous variables were compared using one-way ANOVA or Kruskal–Wallis test as appropriate. Categorical variables were compared using the chi-square test or Fisher’s exact test

**Table 2:** Baseline clinical and OCT characteristics according to long-term anatomical response

Parameter	Full (*n* = 42)	Partial (*n* = 80)	Non (*n* = 42)	*p*
CMT (µm)	433.6 ± 149.7	494.3 ± 133.1	413.5 ± 128.9	0.001
BCVA (logMAR)	0.40 ± 0.38	0.47 ± 0.42	0.42 ± 0.37	0.745
SFCT (µm)	303.0 ± 66.8	318.8 ± 91.5	322.3 ± 99.1	0.556
HRF	6.3 ± 4.8	8.4 ± 6.4	8.2 ± 6.2	0.165
DRIL n (%)	28 (66.7%)	63 (78.8%)	19 (45.2%)	0.001
EZ/ELM defect n (%)	19 (45.2%)	53 (66.3%)	14 (33.3%)	0.001
Cyst size				0.010
Small cyst (≤ 100 μm)	13 (30.9%)	6 (7.5%)	12 (28.6%)	
Large cyst (> 100–200 μm)	8 (19.1%)	17 (21.2%)	10 (23.8%)	
Giant cyst (> 200 μm)	21 (50.0%)	57 (71.3%)	20 (47.6%)	
Hyperreflective intraretinal material	3 (7.1%)	20 (25.0%)	8 (19.1%)	0.049

Continuous variables were compared using one-way ANOVA or Kruskal–Wallis test as appropriate. Categorical variables were compared using the chi-square test or Fisher’s exact test

**Table 3:** Central macular thickness, best-corrected visual acuity, subfoveal choroidal thickness, and hyperreflective foci count at baseline, post-loading, and final follow-up (overall cohort)

Parameter	Baseline	Post-loading	Final follow-up	*p* (baseline vs. post-loading)	*p* (post-loading vs. final)	*p* (baseline vs. final)
CMT (µm)	458.1 ± 140.4 (*n* = 164)	382.6 ± 129.0 (*n* = 164)	321.4 ± 102.8 (*n* = 163)	< 0.001	< 0.001	< 0.001
BCVA (logMAR)	0.44 ± 0.40 (*n* = 164)	0.42 ± 0.40 (*n* = 164)	0.36 ± 0.32 (*n* = 164)	0.211	0.002	< 0.001
SFCT (µm)	315.7 ± 87.8 (*n* = 161)	298.3 ± 87.7 (*n* = 156)	283.9 ± 81.4 (*n* = 139)	0.003	0.015	< 0.001
HRF count	7.9 ± 6.0 (*n* = 164)	6.0 ± 4.2 (*n* = 164)	4.1 ± 3.6 (*n* = 163)	< 0.001	< 0.001	< 0.001

Values are mean ± standard deviation. All pairwise p values are pairwise contrasts from a repeated-measures GEE model (Gaussian family, identity link, exchangeable working correlation, clustered by patient, time as a three-level categorical factor), which accounts for both the within-eye correlation across the three visits and the correlation between the two eyes of patients who contributed both eyes; this replaces the naive paired t-test approach used in an earlier draft, which did not account for inter-eye correlation. Sample sizes vary owing to missing SFCT and final-visit measurements in a minority of eyes

Because both eyes from some patients were included, GEE logistic regression models with an exchangeable working correlation structure and robust standard errors were constructed to account for inter-eye correlation. Separate multivariable GEE models were fitted for early anatomical response and long-term anatomical response (at the final follow-up).

Baseline CMT, baseline BCVA, age, and diabetes duration were included in all multivariable models as prespecified covariates. Baseline OCT biomarkers, including subfoveal choroidal thickness, HRF count, DRIL, EZ/ELM defects, cyst size, and hyperreflective intraretinal material, were first evaluated using univariable GEE analyses. Biomarkers with *p* < 0.10 in the univariable analyses were subsequently entered into the corresponding multivariable GEE model. For the early anatomical response model, HRF count, DRIL, EZ/ELM defects, cyst size, and hyperreflective intraretinal material met the predefined inclusion criterion. For the long-term anatomical response model, DRIL, EZ/ELM defects, and cyst size met the predefined inclusion criterion. Subfoveal choroidal thickness was not included in either multivariable model because it did not meet the predefined inclusion criterion. This *p* < 0.10 screening rule was applied only to the OCT biomarkers listed above; demographic and systemic variables (e.g., male sex, which was significantly associated with long-term response in univariable analysis, Table 4) were not eligible for entry into the multivariable models under this prespecified rule.

**Table 4:** Univariable GEE analysis for long-term anatomical response

Variable	OR (95% CI)	*p*	q (FDR)
Age (per 10 years)	1.13 (0.77–1.66)	0.542	0.705
Male sex	2.21 (1.12–4.37)	0.022	0.057
Duration of diabetes (per 5 years)	0.98 (0.93–1.03)	0.341	0.553
Hypertension (yes/no)	0.89 (0.44–1.78)	0.741	0.741
Baseline HbA1c (%)	1.03 (0.86–1.25)	0.718	0.741
Baseline CMT (per 100 μm)	2.30 (1.45–3.66)	< 0.001	0.002
Baseline BCVA (per 0.1 logMAR)	1.05 (0.97–1.14)	0.218	0.472
Baseline SFCT (per 100 μm)	0.92 (0.62–1.34)	0.654	0.741
Baseline HRF count (per lesion)	1.02 (0.96–1.08)	0.533	0.705
Baseline DRIL (yes/no)	2.93 (1.48–5.78)	0.002	0.006
Baseline EZ/ELM defect (yes/no)	3.55 (1.77–7.11)	< 0.001	0.002
Baseline intraretinal cyst size	0.40 (0.26–0.63)	< 0.001	< 0.001
Baseline hyperreflective intraretinal material (yes/no)	1.57 (0.66–3.72)	0.310	0.553

n and events vary by row owing to missing data (complete-case analysis); overall analytic sample *n* = 164 eyes, 97 long-term responders (59.1%). See Methods for missingness by variable

Intraretinal cyst size was entered as an ordinal variable (1 = giant, 2 = large, 3 = small); therefore, higher values indicate smaller cyst size

Adjusted odds ratios (OR) with 95% confidence intervals (CI) were calculated for all multivariable analyses. To reduce the risk of type I error due to multiple comparisons, the Benjamini–Hochberg false discovery rate (FDR) correction was applied separately within each table (Tables 4, 5, 6, and 7 and Supplementary Tables S1–S2, as well as Supplementary Tables S8–S9), treating the set of variables listed in that table as the family of tests; q values were not pooled across tables. We note that applying FDR correction to the covariates of a single, pre-specified multivariable adjustment model (Tables 6 and 7) is not a conventional use of the method, since these covariates were not independent exploratory hypotheses but a fixed adjustment set; the q values reported for Tables 6 and 7 should therefore be interpreted as an exploratory, conservative supplement to the p values rather than as confirmatory, and we regard the Tables 4 and 5 univariable screening analyses as the primary context in which the FDR correction is methodologically appropriate.

**Table 5:** Univariable GEE analysis for early anatomical response

Variable	OR (95% CI)	*p*	q (FDR)
Age (per 10 years)	0.77 (0.51–1.15)	0.199	0.292
Male sex	1.32 (0.66–2.67)	0.432	0.561
Duration of diabetes (per 5 years)	0.99 (0.94–1.03)	0.574	0.678
Hypertension (yes/no)	0.90 (0.47–1.76)	0.766	0.780
Baseline HbA1c (%)	1.12 (0.94–1.34)	0.202	0.292
Baseline CMT (per 100 μm)	1.96 (1.44–2.65)	< 0.001	< 0.001
Baseline BCVA (per 0.1 logMAR)	1.11 (1.02–1.20)	0.016	0.040
Baseline SFCT (per 100 μm)	1.05 (0.72–1.53)	0.780	0.780
Baseline HRF count (per lesion)	1.07 (1.01–1.13)	0.018	0.040
Baseline DRIL (yes/no)	2.74 (1.27–5.93)	0.011	0.040
Baseline EZ/ELM defect (yes/no)	1.97 (0.99–3.90)	0.053	0.099
Baseline intraretinal cyst size	0.49 (0.29–0.82)	0.007	0.040
Baseline hyperreflective intraretinal material (yes/no)	2.67 (1.19–5.96)	0.017	0.040

n and events vary by row owing to missing data (complete-case analysis); overall analytic sample *n* = 164 eyes, 53 early responders (32.3%). See Methods for missingness by variable

Intraretinal cyst size was entered as an ordinal variable (1 = giant, 2 = large, 3 = small); therefore, higher values indicate smaller cyst size

**Table 6:** Multivariable GEE logistic regression for early anatomical response

Variable	OR (95% CI)	*p*	q (FDR)
Baseline CMT (per 100 μm)	1.89 (1.20–2.99)	0.006	0.019
Baseline BCVA (per 0.1 logMAR)	1.00 (0.90–1.11)	0.987	0.987
Age (per 10 years)	0.83 (0.50–1.39)	0.480	0.865
Duration of diabetes (per 5 years)	1.00 (0.95–1.05)	0.954	0.987
Baseline HRF count (per lesion)	1.10 (1.03–1.17)	0.005	0.019
Baseline DRIL (yes/no)	1.56 (0.62–3.89)	0.344	0.773
Baseline EZ/ELM defect (yes/no)	0.88 (0.35–2.20)	0.784	0.987
Baseline intraretinal cyst size (small/large/giant)	0.99 (0.53–1.87)	0.979	0.987
Baseline hyperreflective intraretinal material (yes/no)	3.70 (1.48–9.27)	0.005	0.019

*n* = 145 eyes with complete data for all model covariates; 47 early responders, 98 non-responders (5.2 events per variable)

Intraretinal cyst size was entered as an ordinal variable (1 = giant, 2 = large, 3 = small); therefore, higher values indicate smaller cyst size. Because ordinal coding imposes equal spacing and a linear effect across three qualitatively different categories, we also re-fitted this model with cyst size as a categorical variable (giant cysts as reference): small cysts, OR 1.05 (0.30–3.69) *p* = 0.945; large cysts, OR 0.85 (0.28–2.52), *p* = 0.764 (Supplementary Table S7). Neither category differed significantly from giant cysts, and the similarity of the two estimates does not support a graded, linear trend across categories; neither the ordinal nor the categorical specification showed an independent association between cyst size and early anatomical response

We also examined whether HRF count, entered as a linear term, adequately captured its association with early anatomical response. Adding a quadratic HRF term to the model did not improve fit (*p* = 0.708), and a categorical analysis using tertiles of HRF count (0–5, 6–9, 10–35 lesions) showed an OR of 1.84 (95% CI 0.65–5.20) for the middle versus lowest tertile and 2.11 (95% CI 0.73–6.08) for the highest versus lowest tertile, a pattern consistent with, but underpowered to confirm, a monotonic linear relationship on the log-odds scale. We therefore retained the linear specification for the primary analysis. Collinearity between baseline CMT and the morphological biomarkers was assessed using variance inflation factors (VIF) for the Table 6 covariate set; all VIFs were below 1.7 (baseline CMT VIF 1.67), indicating that multicollinearity did not materially inflate the standard errors of the model coefficients. This is a distinct issue from the mathematical coupling and confounding between baseline CMT and cyst size discussed above: a low VIF indicates that the model could estimate each coefficient with reasonable precision, not that the estimated cyst-size effect is free of confounding by baseline thickness (Pearson *r* = − 0.52 between baseline CMT and ordinal cyst size in this cohort)

**Table 7:** Multivariable GEE logistic regression for long-term anatomical response

Variable	OR (95% CI)	*p*	q (FDR)
Baseline CMT (per 100 μm)	2.25 (1.14–4.42)	0.019	0.132
Baseline BCVA (per 0.1 logMAR)	0.92 (0.82–1.03)	0.165	0.238
Age (per 10 years)	1.63 (0.96–2.75)	0.071	0.238
Duration of diabetes (per 5 years)	0.96 (0.91–1.02)	0.204	0.238
Baseline DRIL (yes/no)	1.36 (0.62–2.96)	0.438	0.438
Baseline EZ/ELM defect (yes/no)	2.00 (0.87–4.58)	0.103	0.238
Baseline intraretinal cyst size (small/large/giant)	0.70 (0.41–1.20)	0.192	0.238

*n* = 146 eyes with complete data for all model covariates; 84 long-term responders, 62 non-responders (8.9 events per variable, using the minority class)

As a sensitivity analysis, one eye per patient was selected to generate an independent dataset. For patients contributing both eyes, the eye with the higher baseline central macular thickness was included. This severity-based selection was chosen as a conservative, deterministic criterion that retains the more severely affected eye per patient, but we note that it is not equivalent to a randomly selected eye and does not necessarily represent the full cohort; to assess whether this choice materially affected the results, we additionally repeated the one-eye-per-patient analysis using a randomly selected eye per patient (fixed random seed = 123 for reproducibility), reported in Supplementary Tables S8 and S9. The multivariable logistic regression analyses were then repeated using this independent dataset to evaluate the robustness of the primary GEE findings.

Because the study aim was framed in prognostic terms, discrimination and calibration were assessed for the primary multivariable models. The area under the receiver operating characteristic curve (AUC) was calculated from the fitted GEE model probabilities, and a 95% confidence interval accounting for inter-eye correlation was obtained by resampling patients (rather than eyes) with replacement 500 times, so that both eyes of a resampled patient were retained together in each bootstrap replicate. Calibration was assessed by regressing the observed outcome on the logit of the predicted probability and reporting the calibration intercept and slope (ideal values 0 and 1, respectively). Optimism-corrected AUC was estimated using Harrell’s bootstrap method (500 replicates): a model was re-fitted in each patient-level bootstrap sample, evaluated on that same sample (bootstrap apparent performance) and on the original sample (test performance), and the average difference (optimism) was subtracted from the apparent AUC in the original sample. Events per variable were calculated as the number of eyes in the minority outcome category divided by the number of model covariates.

## Results

A total of 164 eyes from 105 patients, comprising 41% females and 59% males, were included in the study. All patients had type 2 diabetes mellitus. The mean age was 59.7 ± 9.0 years, the mean duration of diabetes was 12.1 ± 6.7 years, and the mean follow-up duration was 42.5 ± 36.7 months, with a median of 34.5 months (IQR, 20–51 months; range, 6–210 months). The mean HbA1c level was 8.7 ± 2.1%, the mean total cholesterol level was 183.9 ± 47.2 mg/dL, and the mean LDL cholesterol level was 115.7 ± 39.5 mg/dL. Hypertension was present in 49.5% of patients (Table 8). Regarding diabetic retinopathy severity, none of the eyes had mild non-proliferative diabetic retinopathy (NPDR); 78.7% had moderate NPDR, 4.3% had severe NPDR, and 17.0% had quiescent proliferative diabetic retinopathy (PDR). The mean number of intravitreal injections during the follow-up period was 9.3 ± 4.3 per eye (median, 9; range, 3–22). At baseline, 88.4% of eyes were phakic and 11.6% were pseudophakic; among eyes phakic at baseline, 7.6% underwent cataract surgery during follow-up, identified by a change in lens status from phakic to pseudophakic between the baseline and final examinations. Glycemic control was assessed at both time points (mean HbA1c 8.6 ± 2.1% at baseline versus 7.7 ± 1.9% at the final visit, available in 115 and 114 eyes, respectively).

**Table 8:** Baseline demographic and systemic characteristics

Parameter	Value mean ± standard deviation or number (%).
Age (years)	59.7 ± 9.0
Female, n (%)	43 (41.0%)
Male, n (%)	62 (59.0%)
Duration of diabetes (years)	12.1 ± 6.7
Hypertension, n (%)	52 (49.5%)
Antidiabetic treatment	
Oral Antidiabetic Drugs	34 (39.1%)
Insulin	18 (20.7%)
Combined	35 (40.2%)
HbA1c (%)	8.74 ± 2.09
Total cholesterol (mg/dL)	183.9 ± 47.2
LDL cholesterol (mg/dL)	115.7 ± 39.5
Affected eye	
Right	84 (51.2%)
Left	80 (48.8%)

Antidiabetic treatment percentages are calculated among the 87 of 105 patients (82.9%) receiving pharmacological antidiabetic treatment (oral agents, insulin, or combined); the remaining 18 patients (17.1%) were managed with diet and lifestyle modification alone and are not included in this denominator. Age, sex, duration of diabetes, hypertension, HbA1c, and lipid profile are reported at the patient level (*n* = 105); the affected-eye row is reported at the eye level (*n* = 164, i.e., 59 patients contributing both eyes)

Following the loading phase, 10 eyes (6.1%) were classified as full responders, 72 (43.9%) as partial responders, and 82 (50.0%) as non-responders. At the final follow-up visit, the proportion of full responders increased to 42 eyes (25.6%), while 80 eyes (48.8%) were classified as partial responders and 42 eyes (25.6%) remained non-responders (Tables 1 and 2). Among eyes classified as full responders after the loading phase, 90% maintained a full anatomical response at the final visit, whereas 10% shifted to partial response. None became non-responders. Among eyes initially classified as non-responders, 20.7% achieved a full response, 39.0% became partial responders, and 40.2% remained non-responders at the final visit.

Under the binary outcome definition used for the GEE analyses (≥ 20% reduction in CMT from baseline), 53 eyes (32.3%) were classified as early responders and 111 (67.7%) as non-responders; at the final visit, 97 eyes (59.1%) were classified as long-term responders and 67 (40.9%) as non-responders. These binary counts do not correspond one-to-one with the full/partial/non-responder categories described above, because full and partial responder status was assigned by masked graders based on qualitative resolution of intraretinal and subretinal fluid and restoration of the foveal contour, not solely by the quantitative CMT threshold. Consistent with this, among the 111 binary non-responders at the early time point, 18 eyes had nonetheless reached a near-normal post-loading CMT (< 300 μm) and a further 19 fell within a 15–20% reduction band, together accounting for most of the numerical difference between the two classification schemes. We note this discrepancy explicitly because the direction of association for baseline CMT and HRF count differs between the descriptive (Tables 1 and 2) and multivariable binary (Tables 4, 5, 6 and 7) analyses, as discussed further below.

### Group comparisons

In the descriptive group comparisons, age was the only demographic variable that differed significantly among the early anatomical response groups, with older patients more frequently represented in the non-responder group. No significant differences were observed among the groups with respect to sex, diabetes duration, hypertension, HbA1c level, lipid profile, or diabetes treatment modality. At the final follow-up, none of the evaluated demographic or systemic variables differed significantly among the anatomical response groups (Tables 1 and 2).

Mean BCVA did not change significantly after the loading phase (repeated-measures GEE, *p* = 0.211) but improved significantly by the final follow-up (*p* < 0.001). Mean CMT decreased significantly after the loading phase and continued to decrease throughout follow-up (*p* < 0.001 for all pairwise contrasts). Corresponding values for CMT, BCVA, SFCT, and HRF count at baseline, post-loading, and final follow-up, together with pairwise contrasts from the repeated-measures model, are reported in Table 3. Total intravitreal injection counts (three loading injections plus subsequent PRN injections) were available for 150 of 164 eyes (91.5%) and were compared by response group. Early responders received fewer total injections than non-responders (mean 8.3 ± 3.6, median 8 [IQR 6–10] vs. mean 9.7 ± 4.6, median 9 [IQR 6–12]; Mann-Whitney *p* = 0.109), a difference that did not reach statistical significance but is directionally consistent with non-responding eyes receiving more rescue treatment. Long-term responders and non-responders received a similar number of injections (mean 9.3 ± 4.3 vs. 9.2 ± 4.5; *p* = 0.914). Adding total injection count as a covariate to the long-term multivariable model (*n* = 133 with complete data) did not materially change the results (injection count: OR 0.99, 95% CI 0.90–1.08, *p* = 0.781; baseline CMT remained significant, OR 2.10, 95% CI 1.14–3.88, *p* = 0.017).

Mean SFCT also decreased significantly during follow-up. However, no significant differences in baseline SFCT were observed among the early or long-term anatomical response groups (Tables 1 and 2). Likewise, baseline hard exudates did not differ significantly among the response groups at either time point.

The mean number of HRF decreased significantly during follow-up. Although baseline HRF counts did not differ significantly among the response groups, numerically lower HRF counts were observed in the full responder group.

At baseline, DRIL was present in 67.1% of eyes, whereas EZ/ELM defects were identified in 52.4%. Significant differences among the response groups were observed for both biomarkers in the descriptive comparisons (Tables 1 and 2).

Baseline intraretinal cyst size differed significantly among the early anatomical response groups, with smaller cysts being more frequently observed in full responders. In contrast, hyperreflective intraretinal material did not differ significantly among the early anatomical response groups but differed significantly among the long-term anatomical response groups (Tables 1 and 2).

### Univariable GEE analyses

To account for inter-eye correlation, GEE logistic regression analyses were performed. In the univariable analyses, higher baseline CMT, poorer baseline BCVA, higher baseline HRF count, the presence of DRIL, hyperreflective intraretinal material, and larger baseline intraretinal cyst size were significantly associated with early anatomical response (Table 5). For long-term anatomical response, higher baseline CMT, the presence of DRIL (OR 2.93), EZ/ELM defects (OR 3.55), and larger baseline intraretinal cyst size were significantly associated with a higher likelihood of anatomical response in univariable analysis (Table 4); hyperreflective intraretinal material was not significantly associated with long-term response in this analysis. We note that the direction of the univariable DRIL and EZ/ELM associations, and the association between larger rather than smaller cyst size and response, are counter to clinical expectation and to the descriptive pattern in Table 2, where DRIL was present in 78.8% of partial responders versus 45.2% of non-responders; we return to this apparent paradox, and its likely relationship to the baseline-CMT coupling described below, in the Discussion.

### Multivariable GEE analyses

After adjustment for baseline CMT, baseline BCVA, age, diabetes duration, and eligible OCT biomarkers, higher baseline CMT, higher baseline HRF count, and the presence of hyperreflective intraretinal material remained independently associated with early anatomical response, whereas baseline intraretinal cyst size, DRIL, and EZ/ELM defect were not significant (Table 6).

For long-term anatomical response, baseline CMT was nominally associated with outcome after multivariable adjustment (*p* = 0.019) but did not survive correction for multiple testing (q = 0.132); none of DRIL, EZ/ELM defect, or intraretinal cyst size reached statistical significance after adjustment (Table 7).

Sensitivity analyses performed using one eye per patient, with the same covariates as the corresponding primary model (Supplementary Tables S1 and S2), showed consistency with the primary GEE findings for baseline CMT, DRIL, EZ/ELM defect, and intraretinal cyst size, which remained non-significant across eye-selection strategies (Table 6: OR 0.99, *p* = 0.979; S1: OR 0.86, *p* = 0.741), and hyperreflective intraretinal material, which remained nominally significant across eye-selection strategies (S1: OR 5.13, *p* = 0.012, q = 0.104) but did not survive FDR correction in the higher-baseline-CMT-eye analysis; a comparison using a randomly selected eye per patient (Supplementary Tables S8 and S9) confirmed this pattern, with hyperreflective intraretinal material remaining significant (OR 9.79, q = 0.040) and cyst size remaining non-significant (OR 0.75, *p* = 0.515), although point estimates for both biomarkers varied more across the alternative outcome definitions examined in Supplementary Tables S3–S6.

Discrimination and calibration of the primary multivariable models were assessed to evaluate their prognostic performance. The early-response model (Table 6, now including hyperreflective intraretinal material as a ninth covariate) had an apparent AUC of 0.809 (patient-cluster bootstrap 95% CI 0.760–0.889) with a calibration intercept of − 0.008 and slope of 0.974 in the derivation sample; after Harrell’s bootstrap optimism correction (500 replicates), the AUC was 0.751, with 47 events and 9 covariates (5.2 events per variable). The long-term response model (Table 7, now including intraretinal cyst size in place of hyperreflective intraretinal material) had an apparent AUC of 0.801 (95% CI 0.723–0.901), a calibration intercept of − 0.002 and slope of 0.911, and an optimism-corrected AUC of 0.757, with 62 non-responders and 7 covariates (8.9 events per variable). Both models therefore showed moderate discrimination after correction for optimism, and events per variable were below the conventional minimum of ten for both models. For transparency and to allow the model to be evaluated or applied by others, the full multivariable equations (on the log-odds, i.e. logit, scale) are reported below. Early response: logit(P) = − 3.906 + 0.637×(baseline CMT/100 µm) + 0.001×(baseline BCVA/0.1 logMAR) − 0.186×(age/10 years) + 0.002×(diabetes duration/5 years) + 0.094×(HRF count) + 0.442×DRIL − 0.128×EZ/ELM defect − 0.009×(cyst size, ordinal) + 1.308×hyperreflective material (DRIL, EZ/ELM defect, and hyperreflective material coded 1 if present, 0 if absent; cyst size coded 1 = giant, 2 = large, 3 = small). Long-term response: logit(P) = − 5.494 + 0.810×(baseline CMT/100 µm) − 0.083×(baseline BCVA/0.1 logMAR) + 0.486×(age/10 years) − 0.037×(diabetes duration/5 years) + 0.308×DRIL + 0.692×EZ/ELM defect − 0.355×(cyst size, ordinal). In both equations, the predicted probability of response is *P* = 1/(1 + e−logit(P)). Given the moderate optimism-corrected discrimination and the internal instability of several component biomarkers described below, these equations are provided for research transparency and external validation rather than as a ready-to-use clinical calculator, and we do not recommend their use for individual patient decision-making at this stage.

## Discussion

In the present study, we evaluated the prognostic value of baseline systemic factors and OCT biomarkers for predicting both post-loading and long-term anatomical response in patients with DME treated with three consecutive intravitreal bevacizumab loading injections followed by a PRN regimen. In the primary multivariable analysis, higher baseline CMT, higher HRF count, and the presence of hyperreflective intraretinal material were independently associated with early anatomical response, whereas intraretinal cyst size, DRIL, and EZ/ELM defect were not. For long-term response, only baseline CMT approached significance in multivariable analysis, and it did not survive correction for multiple testing; none of DRIL, EZ/ELM defect, or intraretinal cyst size reached significance after adjustment. Although DRIL and EZ/ELM defects were associated with treatment response in descriptive and univariable analyses, they did not remain independent predictors after multivariable adjustment at either time point, suggesting that they primarily reflect baseline disease severity rather than independently predicting treatment response.

Vitreoretinal interface abnormalities, such as epiretinal membrane and vitreomacular traction, may influence retinal morphology, intraretinal fluid dynamics, and visual outcomes in diabetic macular edema. Therefore, eyes with these conditions were excluded to minimize potential confounding effects on OCT biomarker assessment and treatment response evaluation [19, 20].

Among demographic and systemic variables, only age differed significantly between post-loading response groups, and this association did not persist after multivariable adjustment for baseline CMT, BCVA, diabetes duration, and OCT biomarkers; no other systemic variable (sex, diabetes duration, hypertension, HbA1c, lipid profile, treatment modality) was significant in either the group comparisons or the multivariable analyses. This is broadly consistent with DRCR Protocol T, where baseline ocular characteristics were stronger predictors of treatment response than systemic variables [21], although lower HbA1c and younger age were associated with greater visual improvement, highlighting the greater prognostic importance of baseline retinal characteristics. Chatziralli et al. similarly found limited influence of systemic metabolic parameters on visual outcome in eyes treated with a dexamethasone implant, a different treatment class, suggesting this pattern may generalize beyond anti-VEGF therapy; discrepancies across studies likely reflect differences in populations, protocols, treatment class, follow-up duration, and statistical methodology [21–23].

The dissociation we observed between anatomical response and functional recovery (Table 3) has been widely reported in DME, reflecting that visual recovery depends not only on edema resolution but also on photoreceptor integrity, retinal ischemia, and neuroretinal damage; anatomical improvement may therefore precede functional recovery, particularly in chronic DME. Sun et al. demonstrated that retinal structural disruption has a greater impact on visual prognosis than retinal thickness alone, while the DRCR Retina Network also showed that anatomical improvement does not necessarily translate into proportional visual gain [23, 24].

Higher baseline CMT remained independently associated with early anatomical response, and was nominally associated with long-term anatomical response without surviving correction for multiple testing (q = 0.132); however, this association must be interpreted with caution. In our multivariable models, the outcome was defined as a percentage reduction in CMT from baseline, which means that baseline CMT appears simultaneously as the denominator of the outcome and as a covariate. This mathematical coupling, together with regression to the mean arising from measurement error in the baseline value, can generate a positive association between baseline CMT and anatomical “response” independent of any underlying biological effect, and represents a more parsimonious explanation than a genuine prognostic effect of baseline retinal thickness. The apparent discrepancy between the descriptive comparisons, in which full responders had the lowest baseline CMT, and the multivariable GEE analyses, in which higher baseline CMT predicted response, is consistent with this artefact rather than with a change in statistical contrast alone. To test this directly, we re-fitted the primary models using two outcome definitions that are not arithmetically tied to baseline CMT: (1) an analysis-of-covariance-type GEE model with post-treatment CMT as a continuous outcome, adjusted for baseline CMT, and (2) a binary outcome based on an absolute post-treatment CMT threshold (< 300 μm) (Supplementary Tables S3–S6). Under both alternative definitions, the association between baseline CMT and anatomical outcome was no longer statistically significant and its point estimate fell below unity (early response: OR 0.65, 95% CI 0.37–1.13, *p* = 0.124; long-term response: OR 0.94, 95% CI 0.68–1.30, *p* = 0.716; ANCOVA-type model, long-term: β -3.0 μm per 100 μm of baseline CMT, 95% CI − 21.7 to + 15.7, *p* = 0.752), in contrast to the strong, significant associations observed under the original percentage-reduction definition (OR 1.89 and 2.25, respectively). Baseline CMT was also strongly correlated with intraretinal cyst size in our cohort (mean baseline CMT 520 μm in eyes with giant cysts versus 349 μm in eyes with small cysts), indicating that the cyst-size association reported above may likewise be partly attributable to this coupling rather than to an independent biological effect. The long-term responder proportion under the original percentage-reduction definition in this cohort (97/164 eyes, 59.1%) falls between the 43.1% reported by Valera-Cornejo et al. and the 65.9% reported by Usui-Ouchi et al. using the same definition [15, 16], which is reassuring. Because several other OCT biomarkers evaluated in this study (DRIL, EZ/ELM disruption, and hyperreflective material) are also correlated with baseline CMT, the same coupling may partly underlie their associations with anatomical response as well. Similar baseline-CMT associations have been reported by Sophie et al., who observed greater reductions in retinal thickness in eyes with higher baseline CMT despite the absence of consistently superior visual outcomes [25, 26]. Together, these findings show that baseline CMT is statistically associated with anatomical response in our dataset, but this association may be driven wholly or partly by the mathematical coupling described above rather than by a genuine biological effect. It should not, at present, be presented as a robust prognostic marker, and it should not be considered a surrogate for functional prognosis.

Although SFCT decreased significantly during follow-up, baseline SFCT was not associated with either early or long-term anatomical response in our cohort. The prognostic role of SFCT in diabetic macular edema remains controversial. Rayess et al. reported that greater baseline SFCT was associated with a more favorable short-term anatomical response to anti-VEGF therapy, whereas subsequent studies failed to demonstrate a consistent relationship between baseline SFCT and treatment outcomes [25, 27].

Although baseline HRF count did not differ significantly in the group comparisons, it remained independently associated with post-loading anatomical response under the original percentage-reduction outcome definition in the multivariable GEE analysis. HRF are considered imaging biomarkers of retinal inflammation and are thought to represent activated microglia and inflammatory cells [28, 29]. Therefore, eyes with a higher baseline HRF burden may represent an inflammatory DME phenotype with greater potential for anatomical response. Its emergence as a predictor only after multivariable adjustment may reflect negative confounding by other baseline retinal characteristics. Schreur et al. similarly reported that eyes with higher baseline HRF count exhibited a greater reduction in central macular thickness following anti-VEGF treatment [30]. HRF count was only weakly correlated with baseline CMT (Pearson *r* = 0.16) and, unlike cyst size and hyperreflective material, did not reverse direction across sensitivity analyses, though it likewise lost significance under both alternative outcome definitions (Supplementary Tables S3 and S5) and requires validation against outcome definitions not tied to baseline thickness.

Baseline DRIL and EZ/ELM defects differed significantly in the group comparisons and were associated with anatomical treatment response in the univariable GEE analyses. However, these associations were no longer significant after adjustment for baseline CMT, BCVA, and other OCT biomarkers. This suggests that both biomarkers primarily reflect baseline retinal damage and disease severity rather than independently predict anatomical treatment response. Previous studies have consistently demonstrated that DRIL and outer retinal integrity are closely associated with visual prognosis. Sun et al. identified DRIL as an important predictor of visual acuity, whereas Maheshwary et al. and Das et al. showed that preserved EZ and ELM integrity is associated with better visual outcomes [24, 31, 32]. Our findings further support the concept that DRIL and EZ/ELM defects are better regarded as markers of retinal structural damage and functional prognosis than as independent predictors of anatomical treatment response.

In our study, baseline intraretinal cyst size was associated with early and long-term anatomical response in univariable analysis, but in the opposite direction to previous reports: giant cysts, rather than small cysts, were associated with a higher likelihood of response, and this association did not persist after multivariable adjustment for early response (OR 0.99, 95% CI 0.53–1.87, *p* = 0.979) or in a categorical re-analysis using giant cysts as the reference category (Supplementary Table S7). As noted above, cyst size was also strongly correlated with baseline CMT in our cohort, providing a plausible explanation for the univariable association via the same mathematical-coupling mechanism described for baseline CMT itself. Previous studies have shown that larger intraretinal cysts are associated with greater outer retinal damage, including ellipsoid zone disruption and outer retinal thinning, whereas smaller cysts are associated with better outcomes [26, 33]. Our finding of the opposite direction is therefore inconsistent with this literature and most parsimoniously explained by confounding via baseline retinal thickness rather than a true, reproducible biological effect; we do not interpret cyst size as an independent predictor of anatomical response in this study.

In our study, hyperreflective intraretinal material was independently associated with a higher likelihood of early anatomical response in both univariable (OR 2.67, q = 0.040) and multivariable analysis (OR 3.70, q = 0.019), an association that survived correction for multiple testing; it was not significantly associated with long-term response in this cohort. This direction is opposite to previous reports linking hyperreflective intraretinal material to poorer anti-VEGF response and greater treatment burden [34, 35]. Possible explanations for this discrepancy include differences in patient populations, hyperreflective-material grading criteria, or treatment regimen (bevacizumab loading followed by PRN dosing with multiple agents, versus the fixed regimens used in some prior studies), or hyperreflective material may act as a marker correlated with other favorable baseline characteristics not fully captured by our adjustment set.

In conclusion, under the original percentage-reduction outcome definition, higher baseline CMT, higher HRF count, and the presence of hyperreflective intraretinal material were independently associated with early anatomical response, whereas intraretinal cyst size was not independently associated after adjustment. For long-term response, no OCT biomarker remained significant after multivariable adjustment and correction for multiple testing. Importantly, the association of baseline CMT was not reproduced using outcome definitions not mathematically tied to baseline thickness, and the associations of HRF count and hyperreflective intraretinal material were likewise not confirmed across alternative outcome definitions. Model discrimination was moderate after optimism correction (AUC 0.751 and 0.757). Overall, these findings suggest that the evaluated OCT biomarkers may provide prognostic information, but their clinical utility requires validation using prespecified outcome definitions, appropriate control of multiple testing, and larger prospective cohorts.

The strengths of this study include the use of GEE to account for inter-eye correlation, the long follow-up period, and the evaluation of both early and long-term anatomical outcomes. Multivariable analyses allowed assessment of independent associations beyond descriptive group comparisons, while sensitivity analyses examined the robustness of biomarker associations across eye-selection strategies and alternative outcome definitions. Reproducibility of OCT biomarker grading was also high, supporting the reliability of the imaging assessments.

In addition, our study population predominantly consisted of eyes with moderate to advanced diabetic retinopathy treated at a tertiary referral center, with no eyes presenting with mild non-proliferative diabetic retinopathy. This may reflect referral patterns and could have introduced selection bias, thereby limiting the generalizability of our findings.

A further limitation is the heterogeneity of follow-up duration used to define long-term response (mean 42.5 ± 36.7 months; median 35.0 months; IQR, 19–52 months; range, 6–210 months), which resulted in different cumulative treatment exposures across eyes. Adjustment for follow-up duration did not materially change the long-term results (OR 0.97 per year, 95% CI 0.87–1.09, *p* = 0.619), but cannot fully eliminate the effect of heterogeneous follow-up. A fixed follow-up window was not feasible because of the limited number of eyes available at specific time points. Injection counts were available for 150 eyes (91.5%) and yielded similar findings in sensitivity analysis. Exclusion of eyes with insufficient follow-up may nevertheless have introduced selection bias.

Reproducibility of OCT biomarker grading was assessed in a randomly selected subset of 30 eyes by two masked observers independently re-grading baseline images. Agreement was substantial to almost perfect for categorical biomarkers (Cohen’s κ 0.77–1.00) and excellent for HRF count and SFCT (ICC 0.97 and 1.00, respectively), with full results provided in Supplementary Table S10. Importantly, cyst-size grading referred to the three-level small/large/giant categorical variable used in the analytical models, rather than continuous cyst diameter. The two biomarkers most sensitive to outcome definition, cyst size and hyperreflective intraretinal material, showed the highest agreement, making unreliable grading an unlikely explanation for their sensitivity in the outcome analyses. This reliability sub-study itself used per-image, shuffled grading; for the full-cohort baseline grading outside this 30-eye subset, however, grading was performed per-patient rather than per-image, which remains a residual masking limitation for the full cohort.

Several ocular and systemic factors that may influence macular thickness and visual acuity during prolonged follow-up were incompletely captured. We recorded baseline lens status, cataract surgery during follow-up, and glycemic control at baseline and the final visit. Adding final HbA1c and interim cataract surgery to the long-term model did not materially alter the results, although this analysis was likely underpowered. Panretinal photocoagulation and macular laser treatment during follow-up were not systematically recorded and therefore could not be included in the analyses.

## Supplementary Information

Below is the link to the electronic supplementary material.

Supplementary Material 1

## Data Availability

The datasets used and/or analyzed during the current study are available from the corresponding author on reasonable request.

## References

[1] Shaw JE, Sicree RA, Zimmet PZ. Global estimates of the prevalence of diabetes for 2010 and 2030. Diabetes Res Clin Pract. 2010;87(1):4–14. 10.1016/j.diabres.2009.10.007 19896746

[2] Browning DJ, Stewart MW, Lee C. Diabetic macular edema: Evidence-based management. Indian J Ophthalmol. 2018;66(12):1736–50. 10.4103/ijo.IJO_1240_18 PMC6256891 30451174

[3] Stefanini FR, Badaró E, Falabella P, Koss M, Farah ME, Maia M. Anti-VEGF for the management of diabetic macular edema. J Immunol Res. 2014;2014:632307. 10.1155/2014/632307 PMC3987934 24741610

[4] Amoaku WM, Ghanchi F, Bailey C, Banerjee S, Banerjee S, Downey L, Gale R, Hamilton R, Khunti K, Posner E, et al. Diabetic retinopathy and diabetic macular oedema pathways and management: UK Consensus Working Group. Eye (Lond). 2020;34(Suppl 1):1–51. 10.1038/s41433-020-0961-6 PMC7337227 32504038

[5] Xie XW, Xu L, Wang YX, Jonas JB. Prevalence and associated factors of diabetic retinopathy. The Beijing Eye Study 2006. Graefes Arch Clin Exp Ophthalmol. 2008;246(11):1519–26. 10.1007/s00417-008-0884-6 18604548

[6] Rubino A, Rousculp MD, Davis K, Wang J, Girach A. Diagnosed diabetic retinopathy in France, Italy, Spain, and the United Kingdom. Prim Care Diabetes. 2007;1(2):75–80. 10.1016/j.pcd.2007.02.002 18632023

[7] Qaum T, Xu Q, Joussen AM, Clemens MW, Qin W, Miyamoto K, Hassessian H, Wiegand SJ, Rudge J, Yancopoulos GD, et al. VEGF-initiated blood-retinal barrier breakdown in early diabetes. Invest Ophthalmol Vis Sci. 2001;42(10):2408–13. 11527957

[8] Zhang J, Zhang J, Zhang C, Zhang J, Gu L, Luo D, Qiu Q. Diabetic Macular Edema: Current Understanding, Molecular Mechanisms and Therapeutic Implications. Cells. 2022;11(21):3362. 10.3390/cells11213362 PMC9655436 36359761

[9] Bressler SB, Qin H, Beck RW, Chalam KV, Kim JE, Melia M, Wells JA. 3rd: Factors associated with changes in visual acuity and central subfield thickness at 1 year after treatment for diabetic macular edema with ranibizumab. Arch Ophthalmol. 2012;130(9):1153–61. 10.1001/archophthalmol.2012.1107 PMC3543147 22965591

[10] Munk MR, Somfai GM, de Smet MD, Donati G, Menke MN, Garweg JG, Ceklic L. The Role of Intravitreal Corticosteroids in the Treatment of DME: Predictive OCT Biomarkers. Int J Mol Sci. 2022;23(14):7585. 10.3390/ijms23147585 PMC9319632 35886930

[11] Hillier RJ, Ojaimi E, Wong DT, Mak MY, Berger AR, Kohly RP, Kertes PJ, Forooghian F, Boyd SR, Eng K, et al. AQUEOUS HUMOR CYTOKINE LEVELS AS BIOMARKERS OF DISEASE SEVERITY IN DIABETIC MACULAR EDEMA. Retina. 2017;37(4):761–9. 10.1097/IAE.0000000000001210 27471825

[12] Shah AR, Yonekawa Y, Todorich B, Van Laere L, Hussain R, Woodward MA, Abbey AM, Wolfe JD. Prediction of Anti-VEGF Response in Diabetic Macular Edema After 1 Injection. J Vitreoretin Dis. 2017;1(3):169–74. 10.1177/2474126416682569 PMC5668872 29104958

[13] Singer MA, Kermany DS, Waters J, Jansen ME, Tyler L. Diabetic macular edema: it is more than just VEGF. F1000Res. 2016;5(F1000 Faculty Rev):1019. 10.12688/f1000research.8265.1 PMC4890310 27303642

[14] Nanji K, Hatamnejad A, Grad J, El-Sayes A, Mihalache A, Gemae M, et al. Visual outcomes associated with optical coherence tomography biomarkers in diabetic macular edema: a systematic review. Surv Ophthalmol. 2026;71(2):289–308. 10.1016/j.survophthal.2025.09.009 40967513

[15] Valera-Cornejo DA, Romero-Morales V, García-Roa M, Ramírez-Neria P, Villalpando-Gómez Y, García-Franco R. Association Between First- and Third-Month Responses to Intravitreal Ranibizumab for Diabetic Macular Edema. J Vitreoretin Dis. 2021;5(2):99–107. 10.1177/2474126420936461 PMC9979050 37009080

[16] Usui-Ouchi A, Tamaki A, Sakanishi Y, Tamaki K, Mashimo K, Sakuma T, Ebihara N. Factors Affecting a Short-Term Response to Anti-VEGF Therapy in Diabetic Macular Edema. Life (Basel). 2021;11(2):83. 10.3390/life11020083 PMC7912394 33503815

[17] Suciu CI, Suciu VI, Nicoara SD. Optical Coherence Tomography (Angiography) Biomarkers in the Assessment and Monitoring of Diabetic Macular Edema. J Diabetes Res. 2020;2020:6655021. 10.1155/2020/6655021 PMC7790580 33490283

[18] Reznicek L, Cserhati S, Seidensticker F, Liegl R, Kampik A, Ulbig M, Neubauer AS, Kernt M. Functional and morphological changes in diabetic macular edema over the course of anti-vascular endothelial growth factor treatment. Acta Ophthalmol. 2013;91(7):e529–536. 10.1111/aos.12153 23647578

[19] Mikhail M, Stewart S, Seow F, Hogg R, Lois N. Vitreomacular interface abnormalities in patients with diabetic macular oedema and their implications on the response to anti-VEGF therapy. Graefes Arch Clin Exp Ophthalmol. 2018;256(8):1411–8. 10.1007/s00417-018-4009-6 PMC6060772 29779188

[20] Kulikov AN, Sosnovskii SV, Berezin RD, Maltsev DS, Oskanov DH, Gribanov NA. Vitreoretinal interface abnormalities in diabetic macular edema and effectiveness of anti-VEGF therapy: an optical coherence tomography study. Clin Ophthalmol. 2017;11:1995–2002. 10.2147/OPTH.S146019 PMC5694203 29180844

[21] Bressler SB, Odia I, Maguire MG, Dhoot DS, Glassman AR, Jampol LM, Marcus DM, Solomon SD, Sun JK. Factors Associated With Visual Acuity and Central Subfield Thickness Changes When Treating Diabetic Macular Edema With Anti-Vascular Endothelial Growth Factor Therapy: An Exploratory Analysis of the Protocol T Randomized Clinical Trial. JAMA Ophthalmol. 2019;137(4):382–9. 10.1001/jamaophthalmol.2018.6786 PMC6459102 30676635

[22] Chatziralli I, Theodossiadis P, Parikakis E, Dimitriou E, Xirou T, Theodossiadis G, Kabanarou SΑ. Dexamethasone intravitreal implant in diabetic macular edema: real-life data from a prospective study and predictive factors for visual outcome. Diabetes Therapy. 2017;8(6):1393–404. 10.1007/s13300-017-0332-x PMC5688995 29110201

[23] Bressler NM, Odia I, Maguire M, Glassman AR, Jampol LM, MacCumber MW, Shah C, Rosberger D, Sun JK. Association Between Change in Visual Acuity and Change in Central Subfield Thickness During Treatment of Diabetic Macular Edema in Participants Randomized to Aflibercept, Bevacizumab, or Ranibizumab: A Post Hoc Analysis of the Protocol T Randomized Clinical Trial. JAMA Ophthalmol. 2019;137(9):977–85. 10.1001/jamaophthalmol.2019.1963 PMC6604439 31246237

[24] Sun JK, Lin MM, Lammer J, Prager S, Sarangi R, Silva PS, Aiello LP. Disorganization of the retinal inner layers as a predictor of visual acuity in eyes with center-involved diabetic macular edema. JAMA Ophthalmol. 2014;132(11):1309–16. 10.1001/jamaophthalmol.2014.2350 25058813

[25] Ashraf M, Souka A, Adelman R. Predicting outcomes to anti-vascular endothelial growth factor (VEGF) therapy in diabetic macular oedema: a review of the literature. Br J Ophthalmol. 2016;100(12):1596–604. 10.1136/bjophthalmol-2016-308388 27231313

[26] Sophie R, Lu N, Campochiaro PA. Predictors of Functional and Anatomic Outcomes in Patients with Diabetic Macular Edema Treated with Ranibizumab. Ophthalmology. 2015;122(7):1395–401. 10.1016/j.ophtha.2015.02.036 25870079

[27] Rayess N, Rahimy E, Ying GS, Bagheri N, Ho AC, Regillo CD, Vander JF, Hsu J. Baseline choroidal thickness as a predictor for response to anti-vascular endothelial growth factor therapy in diabetic macular edema. Am J Ophthalmol. 2015;159(1):85–e9181. 10.1016/j.ajo.2014.09.033 25261844

[28] Bolz M, Schmidt-Erfurth U, Deak G, Mylonas G, Kriechbaum K, Scholda C. Optical coherence tomographic hyperreflective foci: a morphologic sign of lipid extravasation in diabetic macular edema. Ophthalmology. 2009;116(5):914–20. 10.1016/j.ophtha.2008.12.039 19410950

[29] Vujosevic S, Bini S, Torresin T, Berton M, Midena G, Parrozzani R, Martini F, Pucci P, Daniele AR, Cavarzeran F, et al. HYPERREFLECTIVE RETINAL SPOTS IN NORMAL AND DIABETIC EYES: B-Scan and En Face Spectral Domain Optical Coherence Tomography Evaluation. Retina. 2017;37(6):1092–103. 10.1097/IAE.0000000000001304 27668929

[30] Schreur V, Altay L, van Asten F, Groenewoud JMM, Fauser S, Klevering BJ, Hoyng CB, de Jong EK. Hyperreflective foci on optical coherence tomography associate with treatment outcome for anti-VEGF in patients with diabetic macular edema. PLoS ONE. 2018;13(10):e0206482. 10.1371/journal.pone.0206482 PMC6209345 30379920

[31] Maheshwary AS, Oster SF, Yuson RM, Cheng L, Mojana F, Freeman WR. The association between percent disruption of the photoreceptor inner segment-outer segment junction and visual acuity in diabetic macular edema. Am J Ophthalmol. 2010;150(1):63–e6761. 10.1016/j.ajo.2010.01.039 PMC2900476 20451897

[32] Das R, Spence G, Hogg RE, Stevenson M, Chakravarthy U. Disorganization of Inner Retina and Outer Retinal Morphology in Diabetic Macular Edema. JAMA Ophthalmol. 2018;136(2):202–8. 10.1001/jamaophthalmol.2017.6256 PMC5838716 29327033

[33] Gerendas BS, Prager S, Deak G, Simader C, Lammer J, Waldstein SM, Guerin T, Kundi M, Schmidt-Erfurth UM. Predictive imaging biomarkers relevant for functional and anatomical outcomes during ranibizumab therapy of diabetic macular oedema. Br J Ophthalmol. 2018;102(2):195–203. 10.1136/bjophthalmol-2017-310483 28724636

[34] Rana V, Agarwal A, Arora A, Bansal R, Dogra M, Bhadada SK, Singh N, Gupta V. Predicting visual outcomes following anti-vascular endothelial growth factor treatment for diabetic macular edema. Indian J Ophthalmol. 2024;72(Suppl 1):S16–21. 10.4103/IJO.IJO_893_23 PMC10833155 38131536

[35] Venkatesh R, Sangai S, Reddy NG, Sridharan A, Pereira A, Aseem A, Gadde SGK, Yadav NK, Chhablani J. Intracystic hyperreflective material in centre-involving diabetic macular oedema. Graefes Arch Clin Exp Ophthalmol. 2021;259(9):2533–44. 10.1007/s00417-021-05083-6 33710472

