# Supplementary material for: Prognostic value of OCT biomarkers and systemic factors for early and long term anatomical treatment response in diabetic macular edema
Source: BMC Ophthalmol. 2026 Sep 17;26:721. doi: 10.1186/s12886-026-05350-2 (PMC13732979; doi:10.1186/s12886-026-05350-2)
Supplement: Supplementary file 1 [file 12886_2026_5350_MOESM1_ESM.docx]

## Supplementary Tables. All sensitivity analyses (S1–S9) use the same covariate set as the corresponding primary model (Table 6 for early response, Table 7 for long-term response), so that differences in results across tables can be attributed to the outcome definition, eye-selection strategy, or cyst-size coding being varied, rather than to an incidental difference in adjustment covariates.

## Supplementary Table S1: Sensitivity analysis (one eye per patient, eye with higher baseline CMT, multivariable standard logistic regression using the SAME covariate set as the primary Table 6 model) – Early response

| **Variable** | **OR (95% CI)** | **p** | **q (FDR)** |
| --- | --- | --- | --- |
| Baseline CMT (per 100 μm) | 1.59 (0.97–2.62) | 0.068 | 0.204 |
| Baseline BCVA (per 0.1 logMAR) | 0.99 (0.86–1.15) | 0.937 | 0.937 |
| Age (per 10 years) | 0.68 (0.38–1.22) | 0.197 | 0.443 |
| Duration of diabetes (per 5 years) | 1.02 (0.96–1.09) | 0.522 | 0.937 |
| Baseline HRF count (per lesion) | 1.09 (1.00–1.19) | 0.045 | 0.203 |
| Baseline DRIL (yes/no) | 1.23 (0.37–4.02) | 0.738 | 0.937 |
| Baseline EZ/ELM defect (yes/no) | 0.90 (0.29–2.79) | 0.848 | 0.937 |
| Baseline intraretinal cyst size | 0.86 (0.36–2.06) | 0.741 | 0.937 |
| Baseline hyperreflective intraretinal material (yes/no) | 5.13 (1.44–18.25) | 0.012 | 0.104 |

*Model: same 9 covariates as Table 6 (baseline CMT, BCVA, age, diabetes duration, HRF count, DRIL, EZ/ELM defect, intraretinal cyst size, hyperreflective intraretinal material), fitted by standard logistic regression in the one-eye-per-patient dataset (n=105; the eye with higher baseline CMT was retained for patients contributing both eyes). Baseline hyperreflective intraretinal material remained nominally significantly associated with early response (OR 5.13, p=0.012), consistent in direction and significance with Table 6 (OR 3.70), although it did not survive FDR correction in this smaller one-eye-per-patient sample (q=0.104); intraretinal cyst size remained non-significant (OR 0.86, p=0.741), also consistent with Table 6.*

## Supplementary Table S2: Sensitivity analysis (one eye per patient, eye with higher baseline CMT, multivariable standard logistic regression using the SAME covariate set as the primary Table 7 model) – Long term response

| **Variable** | **OR (95% CI)** | **p** | **q (FDR)** |
| --- | --- | --- | --- |
| Baseline CMT (per 100 μm) | 2.28 (1.22–4.25) | 0.010 | 0.068 |
| Baseline BCVA (per 0.1 logMAR) | 0.91 (0.79–1.04) | 0.178 | 0.343 |
| Age (per 10 years) | 1.51 (0.80–2.84) | 0.199 | 0.343 |
| Duration of diabetes (per 5 years) | 0.96 (0.89–1.03) | 0.245 | 0.343 |
| Baseline DRIL (yes/no) | 1.63 (0.55–4.80) | 0.379 | 0.442 |
| Baseline EZ/ELM defect (yes/no) | 2.04 (0.65–6.41) | 0.221 | 0.343 |
| Baseline intraretinal cyst size | 0.94 (0.43–2.05) | 0.875 | 0.875 |

*Model: same 7 covariates as Table 7 (baseline CMT, BCVA, age, diabetes duration, DRIL, EZ/ELM defect, intraretinal cyst size), fitted by standard logistic regression in the one-eye-per-patient dataset (n=105). Baseline CMT remained the only nominally significant covariate (p=0.010) but did not survive FDR correction (q=0.068), consistent with Table 7; intraretinal cyst size remained non-significant, also consistent with Table 7.*

## Supplementary Table S3: Sensitivity analysis using post-loading CMT as a continuous outcome, adjusted for baseline CMT (ANCOVA-type GEE model, not mathematically coupled to baseline thickness; SAME 9 covariates as Table 6) – Early response

| **Variable** | **β or OR (95% CI)** | **p** |
| --- | --- | --- |
| Baseline CMT (per 100 μm) | +45.57 (+21.33 to +69.81) | <0.001 |
| Baseline BCVA (per 0.1 logMAR) | +4.99 (-1.53 to +11.51) | 0.134 |
| Age (per 10 years) | +10.04 (-15.91 to +35.98) | 0.449 |
| Duration of diabetes (per 5 years) | -0.27 (-1.89 to +1.35) | 0.743 |
| Baseline HRF count (per lesion) | -2.32 (-6.40 to +1.76) | 0.265 |
| Baseline DRIL (yes/no) | +4.50 (-23.15 to +32.16) | 0.750 |
| Baseline EZ/ELM defect (yes/no) | -1.00 (-36.11 to +34.12) | 0.956 |
| Baseline intraretinal cyst size | -9.48 (-30.40 to +11.44) | 0.374 |
| Baseline hyperreflective intraretinal material (yes/no) | -32.01 (-74.73 to +10.71) | 0.142 |

*Model: post-loading central macular thickness (µm) as a continuous dependent variable, generalized estimating equations (Gaussian family, exchangeable correlation, patient as cluster), with the same 9 covariates as Table 6. β indicates the estimated change in post-loading CMT (µm) per unit increase in the predictor. n=145 eyes with complete data. Baseline CMT remained strongly associated with post-loading CMT (as expected, since baseline and post-loading thickness are physiologically correlated); none of the OCT biomarkers, including hyperreflective intraretinal material, reached significance in this continuous-outcome formulation, in contrast to their status in Table 6 under the original percentage-reduction outcome.*

## Supplementary Table S4: Sensitivity analysis using final-visit CMT as a continuous outcome, adjusted for baseline CMT (ANCOVA-type GEE model, not mathematically coupled to baseline thickness; SAME 7 covariates as Table 7) – Long term response

| **Variable** | **β or OR (95% CI)** | **p** |
| --- | --- | --- |
| Baseline CMT (per 100 μm) | -3.02 (-21.74 to +15.71) | 0.752 |
| Baseline BCVA (per 0.1 logMAR) | -0.95 (-6.70 to +4.80) | 0.745 |
| Age (per 10 years) | -4.98 (-26.30 to +16.34) | 0.647 |
| Duration of diabetes (per 5 years) | +0.73 (-1.32 to +2.77) | 0.487 |
| Baseline DRIL (yes/no) | +11.14 (-20.56 to +42.84) | 0.491 |
| Baseline EZ/ELM defect (yes/no) | -8.54 (-34.96 to +17.87) | 0.526 |
| Baseline intraretinal cyst size | -14.29 (-32.73 to +4.16) | 0.129 |

*Model: final-visit central macular thickness (µm) as a continuous dependent variable, generalized estimating equations (Gaussian family, exchangeable correlation, patient as cluster), with the same 7 covariates as Table 7. n=145 eyes with complete data. Baseline CMT is no longer associated with final CMT in this formulation (p=0.752), consistent with the loss of the baseline-CMT association under alternative outcome definitions described in the main text; none of DRIL, EZ/ELM defect, or intraretinal cyst size reached significance, consistent with Table 7.*

## Supplementary Table S5: Sensitivity analysis using an absolute post-loading CMT threshold (<300 µm), an outcome not mathematically coupled to baseline CMT (SAME 9 covariates as Table 6) – Early response

| **Variable** | **OR (95% CI)** | **p** |
| --- | --- | --- |
| Baseline CMT (per 100 μm) | 0.65 (0.37–1.13) | 0.124 |
| Baseline BCVA (per 0.1 logMAR) | 0.95 (0.84–1.07) | 0.383 |
| Age (per 10 years) | 1.02 (0.57–1.82) | 0.957 |
| Duration of diabetes (per 5 years) | 0.93 (0.85–1.01) | 0.091 |
| Baseline HRF count (per lesion) | 1.04 (0.96–1.12) | 0.341 |
| Baseline DRIL (yes/no) | 1.03 (0.46–2.31) | 0.949 |
| Baseline EZ/ELM defect (yes/no) | 1.26 (0.50–3.16) | 0.619 |
| Baseline intraretinal cyst size | 1.56 (0.93–2.62) | 0.094 |
| Baseline hyperreflective intraretinal material (yes/no) | 2.25 (0.70–7.16) | 0.172 |

*Model: binary outcome defined as post-loading CMT <300 µm (vs. ≥300 µm), generalized estimating equations (binomial family, exchangeable correlation, patient as cluster), with the same 9 covariates as Table 6. OR (95% CI). n=145 eyes with complete data. Baseline CMT is no longer a significant predictor under this outcome definition and its point estimate falls below 1; hyperreflective intraretinal material is directionally consistent with Table 6 (OR>1) but no longer reaches significance (p=0.172), illustrating outcome-definition sensitivity even for this otherwise robust biomarker.*

## Supplementary Table S6: Sensitivity analysis using an absolute final-visit CMT threshold (<300 µm), an outcome not mathematically coupled to baseline CMT (SAME 7 covariates as Table 7) – Long term response

| **Variable** | **OR (95% CI)** | **p** |
| --- | --- | --- |
| Baseline CMT (per 100 μm) | 0.94 (0.68–1.30) | 0.716 |
| Baseline BCVA (per 0.1 logMAR) | 0.99 (0.88–1.11) | 0.859 |
| Age (per 10 years) | 1.04 (0.66–1.65) | 0.861 |
| Duration of diabetes (per 5 years) | 0.99 (0.94–1.04) | 0.612 |
| Baseline DRIL (yes/no) | 0.94 (0.42–2.11) | 0.890 |
| Baseline EZ/ELM defect (yes/no) | 3.44 (1.57–7.52) | 0.002 |
| Baseline intraretinal cyst size | 1.31 (0.78–2.19) | 0.307 |

*Model: binary outcome defined as final-visit CMT <300 µm (vs. ≥300 µm), generalized estimating equations (binomial family, exchangeable correlation, patient as cluster), with the same 7 covariates as Table 7. OR (95% CI). n=146 eyes with complete data. Baseline CMT is no longer a significant predictor under this outcome definition. EZ/ELM defect becomes strongly significant here (OR 3.44, p=0.002) despite being non-significant in Table 7, illustrating that this association is highly sensitive to the outcome definition used and should not be regarded as an established, outcome-independent finding.*

## Supplementary Table S7: Sensitivity analysis with intraretinal cyst size entered as a categorical variable (giant cysts as reference) instead of ordinal (SAME covariates as Table 6, cyst size recoded) – Early response, multivariable GEE model

| **Variable** | **OR (95% CI)** | **p** |
| --- | --- | --- |
| Baseline CMT (per 100 μm) | 1.86 (1.17–2.96) | 0.009 |
| Baseline BCVA (per 0.1 logMAR) | 1.00 (0.90–1.12) | 0.950 |
| Age (per 10 years) | 0.83 (0.49–1.39) | 0.472 |
| Duration of diabetes (per 5 years) | 1.00 (0.95–1.05) | 0.990 |
| Baseline HRF count (per lesion) | 1.10 (1.03–1.17) | 0.004 |
| Baseline DRIL (yes/no) | 1.56 (0.63–3.89) | 0.334 |
| Baseline EZ/ELM defect (yes/no) | 0.88 (0.35–2.21) | 0.787 |
| Small cyst (vs. giant, reference) | 1.05 (0.30–3.69) | 0.945 |
| Large cyst (vs. giant, reference) | 0.85 (0.28–2.52) | 0.764 |
| Baseline hyperreflective intraretinal material (yes/no) | 3.77 (1.47–9.68) | 0.006 |

*Model: same covariates and outcome as Table 6, but intraretinal cyst size entered as a categorical variable with giant cysts as the reference category, instead of the ordinal coding used in the primary analysis. n=145 eyes with complete data. Neither small nor large cysts differed significantly from giant cysts, consistent with the null ordinal finding in Table 6 (OR 0.99, p=0.979); hyperreflective intraretinal material remained strongly significant (OR 3.77, p=0.006) in this alternative specification, further supporting the robustness of this association to how cyst size is coded.*

## Supplementary Table S8: Sensitivity analysis (one eye per patient, RANDOMLY selected eye, multivariable standard logistic regression using the SAME covariate set as the primary Table 6 model) – Early response

| **Variable** | **OR (95% CI)** | **p** | **q (FDR)** |
| --- | --- | --- | --- |
| Baseline CMT (per 100 μm) | 1.49 (0.89–2.50) | 0.128 | 0.384 |
| Baseline BCVA (per 0.1 logMAR) | 0.99 (0.85–1.15) | 0.880 | 0.980 |
| Age (per 10 years) | 0.86 (0.47–1.57) | 0.617 | 0.793 |
| Duration of diabetes (per 5 years) | 1.03 (0.96–1.09) | 0.423 | 0.793 |
| Baseline HRF count (per lesion) | 1.13 (1.03–1.23) | 0.009 | 0.040 |
| Baseline DRIL (yes/no) | 1.40 (0.44–4.47) | 0.566 | 0.793 |
| Baseline EZ/ELM defect (yes/no) | 1.01 (0.31–3.33) | 0.980 | 0.980 |
| Baseline intraretinal cyst size | 0.75 (0.32–1.77) | 0.515 | 0.793 |
| Baseline hyperreflective intraretinal material (yes/no) | 9.79 (1.95–49.15) | 0.006 | 0.040 |

*One eye per patient was selected at random (fixed seed = 123) rather than by baseline severity, using the same 9 covariates as Table 6, for comparison with Supplementary Table S1. n=105 patients. Baseline hyperreflective intraretinal material remained significant regardless of eye-selection strategy (OR 9.79 here, q=0.040, vs. OR 5.13 in S1, q=0.104, vs. OR 3.70 in Table 6, q=0.019); the association survived FDR correction in this random-eye analysis and in the primary Table 6 analysis, though not in the higher-baseline-CMT-eye analysis (S1), supporting the overall robustness of this association while noting some sensitivity to eye-selection at the level of the FDR-corrected q-value specifically. Intraretinal cyst size remained non-significant across all three eye-selection strategies (Table 6: OR 0.99, p=0.979; S1: OR 0.86, p=0.741; S8: OR 0.75, p=0.515), consistently supporting a null finding for this biomarker across eye-selection strategies, although its point estimate varies more across the alternative outcome definitions examined in Supplementary Tables S3–S6.*

## Supplementary Table S9: Sensitivity analysis (one eye per patient, RANDOMLY selected eye, multivariable standard logistic regression using the SAME covariate set as the primary Table 7 model) – Long term response

| **Variable** | **OR (95% CI)** | **p** | **q (FDR)** |
| --- | --- | --- | --- |
| Baseline CMT (per 100 μm) | 2.31 (1.25–4.24) | 0.007 | 0.051 |
| Baseline BCVA (per 0.1 logMAR) | 0.94 (0.82–1.07) | 0.354 | 0.413 |
| Age (per 10 years) | 1.95 (1.02–3.73) | 0.043 | 0.150 |
| Duration of diabetes (per 5 years) | 0.96 (0.89–1.03) | 0.262 | 0.397 |
| Baseline DRIL (yes/no) | 1.25 (0.43–3.58) | 0.681 | 0.681 |
| Baseline EZ/ELM defect (yes/no) | 1.85 (0.60–5.72) | 0.283 | 0.397 |
| Baseline intraretinal cyst size | 0.65 (0.32–1.33) | 0.242 | 0.397 |

*One eye per patient was selected at random (fixed seed = 123) rather than by baseline severity, using the same 7 covariates as Table 7, for comparison with Supplementary Table S2. n=105 patients. Baseline CMT and intraretinal cyst size are directionally consistent with Table 7 and S2 across all eye-selection strategies (CMT nominally significant but not surviving FDR; cyst size non-significant throughout); age reaches nominal significance here (p=0.043) but not in S2 or Table 7, illustrating modest sampling variability with the smaller one-eye-per-patient sample.*

## Supplementary Table S10: Inter-observer reliability for OCT biomarker grading, randomly selected subset of 30 eyes

| **Variable** | **Statistic** | **Estimate** | **95% CI / Limits of agreement** |
| --- | --- | --- | --- |
| DRIL (present/absent) | Cohen's kappa | 0.77 | 0.53 to 1.00 |
| EZ/ELM defect (present/absent) | Cohen's kappa | 0.78 | 0.54 to 1.00 |
| Hyperreflective intraretinal material (present/absent) | Cohen's kappa | 1.00 | 1.00 to 1.00 |
| Intraretinal cyst size (category: small/large/giant) | Cohen's kappa | 0.95 | 0.86 to 1.00 |
| HRF count | ICC (2,1) | 0.97 | Bland-Altman mean diff. −0.07 lesions, LoA −2.18 to +2.05 |
| SFCT (μm) | ICC (2,1) | 1.00 | Bland-Altman mean diff. −0.47 µm, LoA −6.15 to +5.21 |

*Two masked observers independently re-graded baseline OCT images for 30 randomly selected eyes (fixed random seed=2026), presented in a shuffled, de-identified order with no access to follow-up images or knowledge of outcome status. Kappa values are interpreted per Landis and Koch (0.61–0.80 = substantial agreement, 0.81–1.00 = almost perfect agreement); ICC values above 0.90 indicate excellent reliability. Note that reproducibility was assessed for intraretinal cyst size as the three-level categorical variable used in the analytical models (small/large/giant), not as continuous diameter in micrometers. Intraretinal cyst size and hyperreflective intraretinal material, the two biomarkers whose point estimates varied most across the alternative outcome definitions examined in Supplementary Tables S3–S6, showed the highest measured agreement of all variables assessed here (cyst-size category itself was consistently non-significant, and hyperreflective intraretinal material consistently significant, across eye-selection strategies, as detailed in Supplementary Tables S1, S7, and S8), indicating that grading unreliability is unlikely to explain the sensitivity of these associations to outcome definition.*
